# Supplementary material for: Generating viable mice with heritable embryonically lethal mutations using the CRISPR-Cas9 system in two-cell embryos
Source: Nat Commun. 2019 Jun 28;10:2883. doi: 10.1038/s41467-019-10748-2 (PMC6599060; doi:10.1038/s41467-019-10748-2)
Supplement: Supplementary file 1 — Supplementary information [file 41467_2019_10748_MOESM1_ESM.pdf]

Generating viable mice with heritable embryonically lethal mutations using the CRISPR/Cas9 system in two-cell embryos Yi Wu *et al.*

Supplemental Information includes figures, tables, and one movie.

Supplementary figure 1. Effects of different concentrations of Cas9 mRNA and sgRNA, with and without supplementation of wild-type (wt) donor oligo, on production of wt alleles of the ten-eleven translocation 2 (*Tet2*) gene.

Supplementary Figure 2. Summary of quality control analyses of whole-genome sequencing data from six mice (sgRNA two-cell embryo #10, #17, and #18 and wild-type (wt) #1, #2, #3).

Supplementary figure 3. Hematoxylin/erythrosine stained tissues from the kidney, lung, liver, submandibular gland, brain, spleen, heart, and thymus of both wild-type (wt) and *Virma* mutant (ko-two-cell embryo) mice generated by microinjection into one blastomere of two-cell embryos.

Supplementary figure 4. *Virma* coverage plot: the coverage of mapped reads from five mice (sgRNA1 two-cell embryo #9 and #11, wild-type (wt) #1, #2, #3) using RNA deep sequencing on the *Virma* locus is shown.

Supplementary table 1. Effect of different concentrations of Cas9 mRNA, sgRNA, and donor oligo on embryo survival based on blastocyst development.

Supplementary table 2. Summary of 34 offspring (F2) of the F1 crossing, *Virma*$^{+/-}$ × *Virma*$^{+/-}$, showing actual inheritance and expected Mendelian inheritance.

Supplementary table 3. Results of CRISPR/Cas9-mediated *Slc17a5* or *Ctla-4* targeting in C57BL/6J mice produced by microinjection into one blastomere of two-cell embryos derived from *in vitro* fertilization.

Supplementary table 4. Summary of all single-nucleotide variants detected in whole-genome sequencing data from three mutant mice (#10, #17, and #18).

Supplementary table 5. Summary of differentially expressed genes (fold change > 1.5 or < 0.5) identified by RNA-seq of kidney tissue from both wt mice and *Virma* mutant mice.

Supplementary table 6. Oligonucleotides used in this study, related to experimental procedures.

Supplementary table 7. Reagents used in this study, related to experimental procedures.

Supplementary movie 1 Phenotype of founder chimeric *Slc17a5* knockout mice generated by microinjecting Cas9 mRNA and sgRNA into zygotes. Founder chimeric mice with the *Slc17a5* knockout developed a severe tremor and uncoordinated gait, appeared weak, and typically died during the third postnatal week.

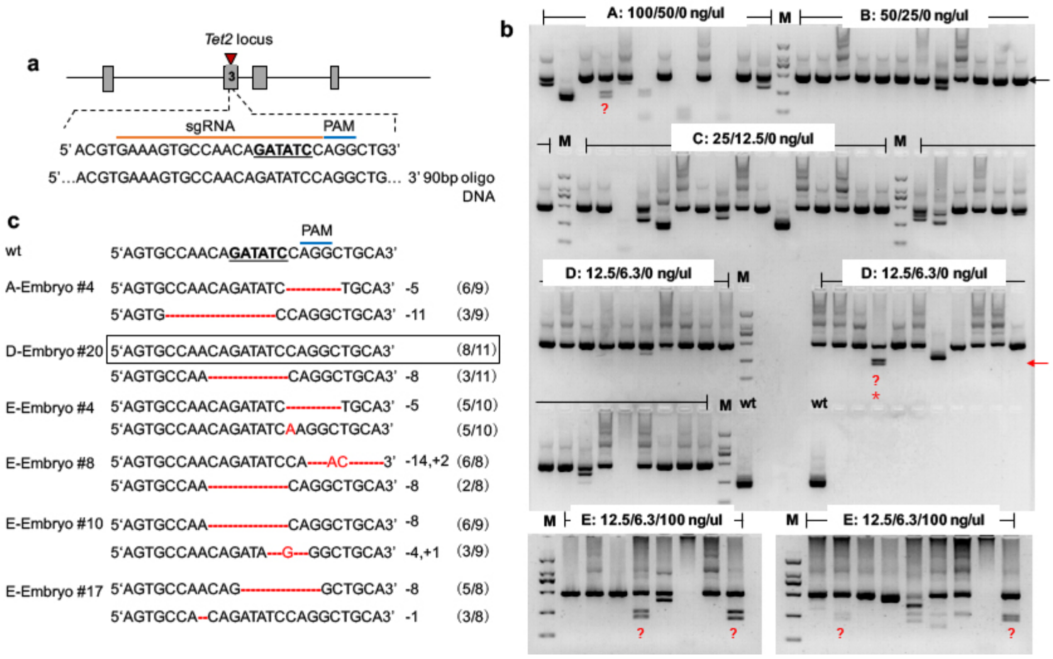

Supplementary figure 1. Effects of different concentrations of Cas9 mRNA and sgRNA, with and without supplementation of wild-type (wt) donor oligo, on production of wt alleles of the ten-eleven translocation 2 (*Tet2*) gene. a Schematic diagram of sgRNA targeting site in the mouse *Tet2* locus. The sgRNA-targeting sequence is underlined, and the protospacer adjacent motif (PAM) sequence is labeled in green. Restriction enzyme site for *EcoR*V used for restriction fragment length polymorphism (RFLP) analysis is bold and capitalized. **b** Genotypes of the *Tet2* locus in mouse blastocysts using RFLP analysis. The cleaved band from the *EcoR*V site is indicated by a red arrow; the band without cleavage by *EcoR*V is indicated by a black arrow. The possible samples with wt alleles are indicated by a red question mark (?), and the sample with a wt allele, confirmed by individual clone sequencing, is indicated by a red asterisk (*). **c** The possible samples with wt alleles were subcloned to determine individual alleles. The boxed sequence indicated a wt allele.

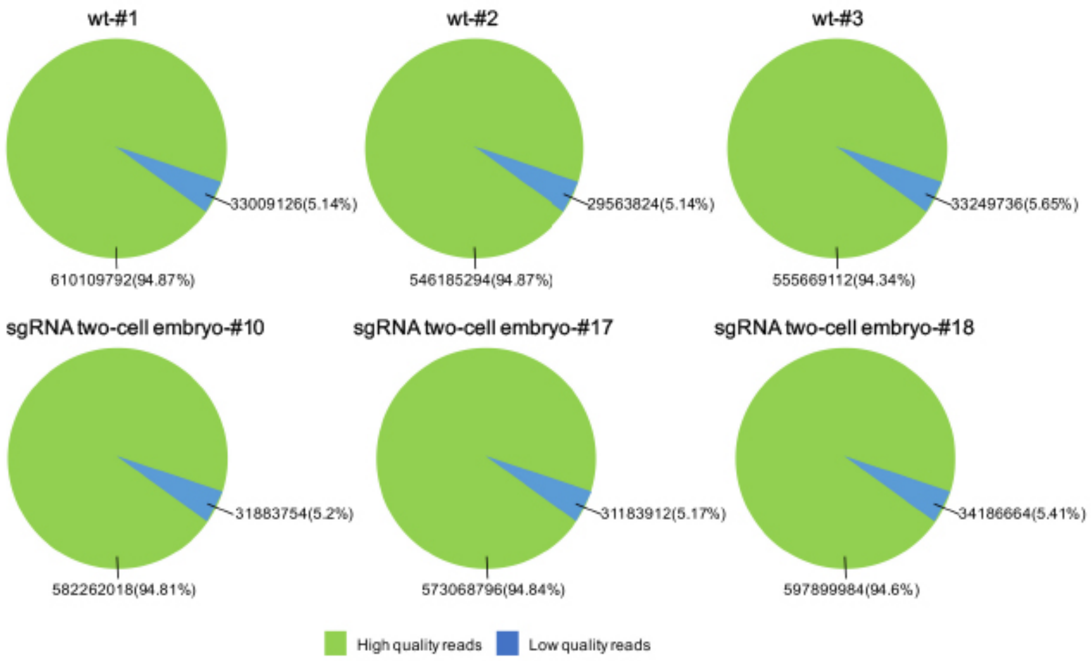

Supplementary Figure 2. Summary of quality control analyses of whole-genome sequencing data from six mice (sgRNA two-cell embryo #10, #17, and #18 and wild-type (wt) #1, #2, #3).

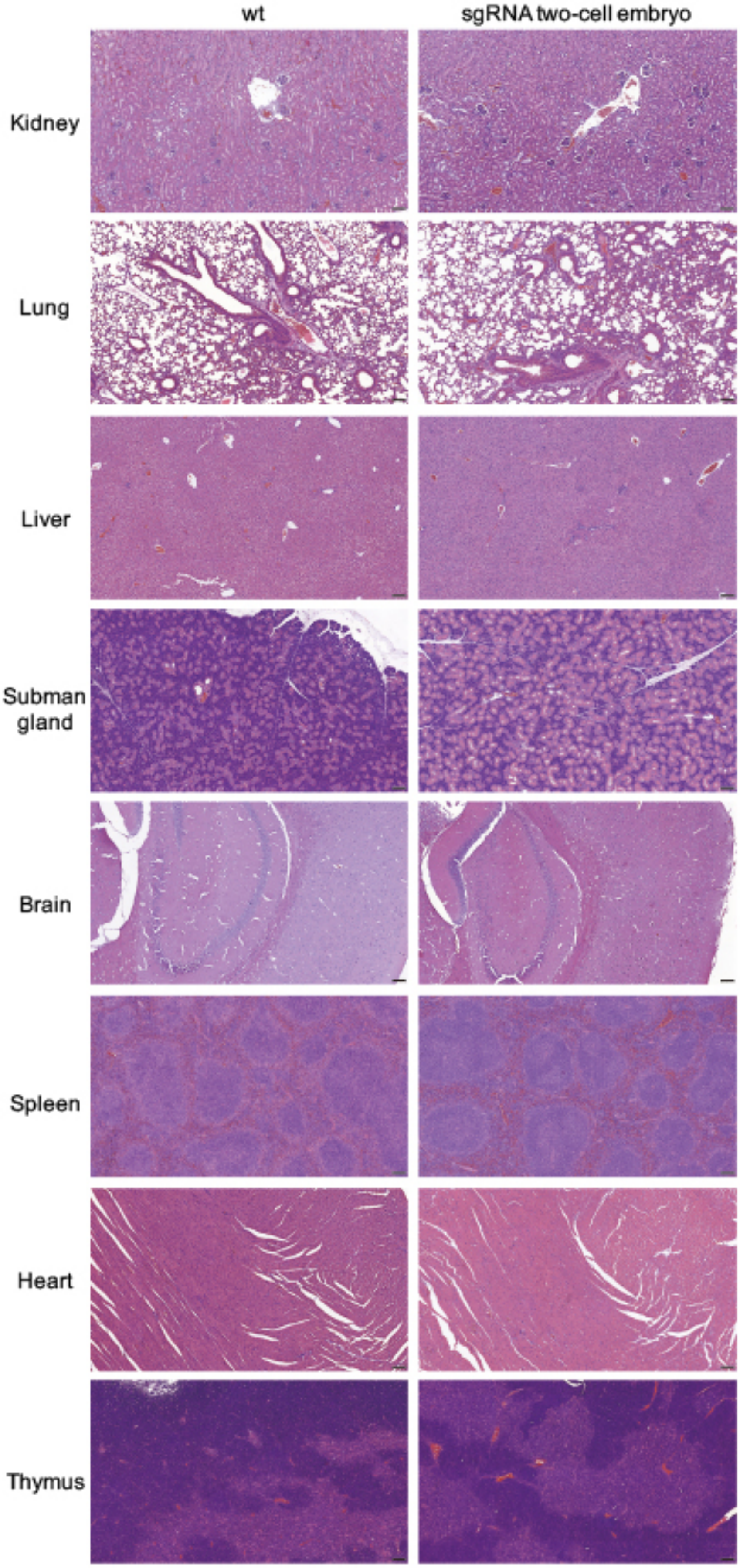

wt
sgRNA two-cell embryo
Kidney
Lung
Liver
Subman gland
Brain
Spleen
Heart
Thymus

Supplementary figure 3. Hematoxylin/erythrosine stained tissues from the kidney, lung, liver, submandibular gland, brain, spleen, heart, and thymus of both wild-type (wt) and *Virma* mutant (ko-two-cell embryo) mice generated by microinjection into one blastomere of two-cell embryos. Scale bar: 100 µm.

Coverage

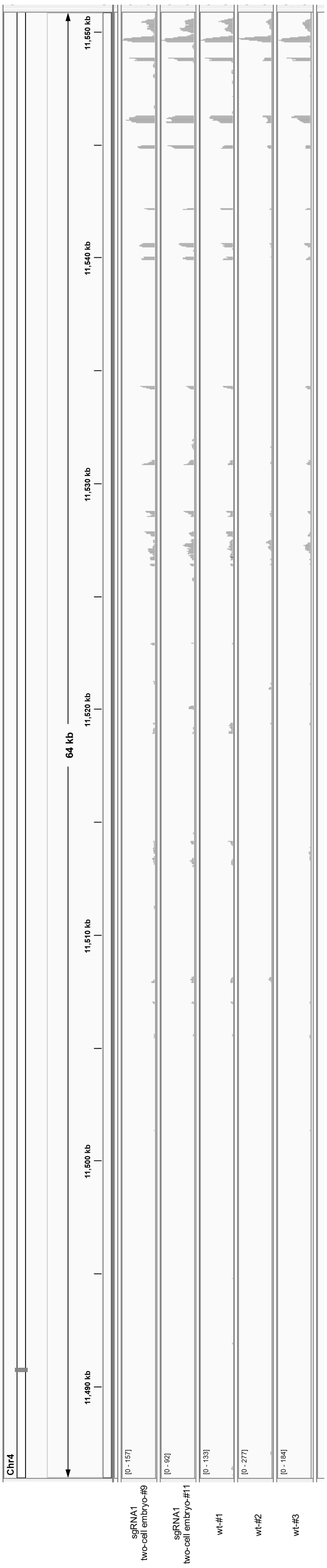

Chr4
64 kb
11,490 kb
11,500 kb
11,510 kb
11,520 kb
11,530 kb
11,540 kb
11,550 kb
[0 - 157]
[0 - 92]
[0 - 133]
[0 - 277]
[0 - 184]
sgRNA1
two-cell embryo-#9
sgRNA1
two-cell embryo-#11
wt-#1
wt-#2
wt-#3

Supplementary figure 4. *Virma* coverage plot: the coverage of mapped reads from five mice (sgRNA1 two-cell embryo #9 and #11, wild-type (wt) #1, #2, #3) using RNA deep sequencing on the *Virma* locus is shown.

Groups	Cas9/sgRNA/donor oligo conc. (ng/ul)	No. of injected embryos	No. of blastocysts developed	Rates of blastocysts developed (%)	NHEJ Efficiency (%)
A	100/50/0	35	12	34.3[a]	70
B	50/25/0	26	13	50.0[a]	100
C	25/12.5/0	34	17	50.0[a]	87
D	12.5/6.3/0	49	36	73.5[b]	97
E	12.5/6.3/100	38	17	44.7[a]	100

Supplementary table 1. Effect of different concentrations of Cas9 mRNA, sgRNA, and donor oligo on embryo survival based on blastocyst development. NHEJ is non-homologous end joining. [a-b] Values with single superscripts differ significantly (chi-squared test, $P < 0.05$)

Virma	+/+	+/-	-/-
Observed (n)	11	23	0
Expected (n)	8.5	17	8.5

Supplementary table 2. Summary of 34 offspring (F2) of the F1 crossing, *Virma*$^{+/-}$ × *Virma*$^{+/-}$, showing actual inheritance and expected Mendelian inheritance.

Gene	Group	Embryos transferred (recipients) (n)	Newborns (n)	Mutant mice (n)	NHEJ efficiency (%)	Mice carrying frameshift mutations (n)	Mice carrying a clear frameshift mutation (n)	Surviving adult mutant mice (n)
Slc17a5	**sgRNA two-cell embryo**	97(4)	45	18	40	11	3	11
Ctla-4	**sgRNA two-cell embryo**	50(2)	15	12	80	8	3	7

Supplementary table 3. Results of CRISPR/Cas9-mediated *Slc17a5* or *Ctla-4* targeting in C57BL/6J mice produced by microinjection into one blastomere of two-cell embryos derived from *in vitro* fertilization. NHEJ = non-homologous end joining.

Chromosome	Position	Reference	Alternative	Gene	Type
NC_000069.6	25961922	G	A	Nlgn1	intron_variant
NC_000071.6	110583667	G	A	Galnt9	intron_variant
NC_000071.6	119225405	C	T	id369953-id369960	intergenic_region
NC_000076.6	7281224	C	A	id673944-Gene_gene24540	intergenic_region
NC_000076.6	11811734	G	A	Gene_gene24585-id675387	intergenic_region
NC_000082.6	30264723	T	TGAAA	Lrrc15	downstream_gene_variant

Supplementary table 4. Summary of all single-nucleotide variants detected in whole-genome sequencing data from three mutant mice (#10, #17, and #18).

Gene	Mean expression of wt	Mean expression of knockout	Change Folds (knockout vs. wt)	P values	Gene ID.
Adra2b	35.8	54.7	1.6	0.03	11552
Alas2	2.3	5.1	2.4	0.02	11656
Apom	57.8	108.9	2.1	<0.01	55938
Car9	2.4	4.5	1.9	0.03	230099
Cyp24a1	23.0	35.2	1.5	0.04	13081
Dgkd	4.9	7.8	1.6	0.045	227333
Etnppl	1.1	2.8	2.2	0.02	71760
Folr1	129.6	192.4	1.6	0.04	14275
Il5ra	0.2	0.7	3.6	<0.01	16192
Kcnk5	35.4	60.6	1.7	<0.01	16529
Mapk14	18.6	28.5	1.6	0.04	26416
Narf	4.2	7.0	1.6	0.03	67608
Rnf186	25.7	39.1	1.6	0.03	66825
Slc17a4	0.5	1.2	2.2	0.04	319848
Slc6a19	31.1	49.1	1.5	0.04	74338
Spata5l1	2.4	5.0	2.5	0.04	214616
Cfd	56.6	3.3	0.08	0.04	11537
Chrnb1	2.9	1.4	0.5	0.04	11443
Cish	8.8	2.7	0.3	<0.01	12700
Cyr61	32.6	12.8	0.4	0.02	16007
Foxs1	4.9	2.0	0.5	0.04	14239
Gm40055	0.3	0.1	0.3	0.04	105244449
Mamdc4	0.3	0.1	0.4	0.04	381352
Noxo1	0.8	0.2	0.3	0.02	71893
Pla2g12b	0.7	0.1	0.1	<0.01	69836
Rasd1	12.1	3.5	0.3	0.02	19416
Tnc	1.3	0.5	0.4	0.04	21923
Virma	2.4	3.0	1.2	0.4	66185

Supplementary table 5 Summary of differentially expressed genes (fold change > 1.5 or < 0.5) identified by RNA-seq of kidney tissue from both wt mice and *Virma* mutant mice.

Supplementary table 6. Oligonucleotides used in this study, related to experimental procedures.

Oligonucleotides used for making template for in vitro transcription.

Template	Direction	Sequence (5' to 3')
Slc17a5 sgRNA	F	taatacgactcactatagGGGCGCCCGGCAGACCGAAGgtttaagagctatgctggaaa
	R	aaaagcaccgactcggtgcc
Virma sgRNA1	F	taatacgactcactatagCTATGGGCTCGTACTCCCGGgtttaagagctatgctggaaa
	R	aaaagcaccgactcggtgcc
Virma sgRNA2	F	taatacgactcactatagGAAGTCCGAGTTATACCCCCgtttaagagctatgctggaaa
	R	aaaagcaccgactcggtgcc
Virma sgRNA3	F	taatacgactcactatagTGAACTGCTAGACCTGTATCgtttaagagctatgctggaaa
	R	aaaagcaccgactcggtgcc
Dpm1 sgRNA	F	taatacgactcactatagGAACAACTGGCAGAGATCTAgtttaagagctatgctggaaa
	R	aaaagcaccgactcggtgcc
Ctla-4 sgRNA	F	taatacgactcactatagGATGAAAAGAAGAGTGAGCAgtttaagagctatgctggaaa
	R	aaaagcaccgactcggtgcc
Tet2 sgRNA	F	taatacgactcactatagGAAAGTGCCAACAGATATCCgtttaagagctatgctggaaa
	R	aaaagcaccgactcggtgcc

Cas9	F	TAATACGACTCACTATAGGGAGAATGGACTATAAGGACCACGAC
	R	GCGAGCTCTAGGAATTCTTAC

Oligonucleotides used for DNA genotyping of mouse tail.

Template	Direction	Sequence (5’ to 3’)
Slc17a5	F	CAACAAGGCGCTACCAGATCA
	R	CACGGCGAAGGGCTGTCCTGC
Virma	F	CCCTGGATTTTAAGATTGTTGGCT
	R	TCCACCCTGAATCTGTCCCATA
Dpm1	F	ACAAAGCCAGAGGTCAACCAA
	R	GGGCATTATGGTGGTAGCGG
Ctla-4	F	AGCTAAACCCACGGCTTCC
	R	GCTCCTTCGCTACTGCTAGA

Oligonucleotides used for DNA genotyping of mouse blastocyst.

Template	Direction	Sequence (5’ to 3’)
Tet2 1st	F	GACCCCTCCTCAGAAGGACA
	R	TGGATCCCAGACTCCAGCTTA
Tet2 2st	F	CAGATGCTTAGGCCAATCAAG
	R	AGAAGCAACACACATGAAGATG

Oligonucleotides used for single cell PCR.

Template	Direction	Sequence (5’ to 3’)
Ctla-4 1st	F	AACAGCTAAACCCACGGCT
	R	GCTCCTTCGCTACTGCTAGA

Ctla-4 2st	F	AGCTAAACCCACGGCTTCC
	R	CTTCGCTACTGCTAGACCTCC

Oligonucleotides used for q-PCR of *Slc17a5* and *Virma* mRNA.

Template	Direction	Sequence (5' to 3')
Slc17a5	F	GGGCGCCCGGCAGACCGAAG
	R	CAACACTCAGGTTCACCCGT
Virma	F	CCCGGGAGTACGAGCCC
	R	TCATCATATTCCAGGCTTCCCA
Gapdh	F	TCACCACCATGGAGAAGGC
	R	GCTAAGCAGTTGGTGGTGCA

Oligonucleotides used for HDR-mediated repair through zygote injection.

Target gene	Sequence (5' to 3')
Tet2	TGCAGCTCGGGCGTGGTTCTCCGATTCTGCAGCCTGG ATATCTGTTGGCACTTTCACGTGTTTCTGTGACTTGAG AGTCAGAGCCTTATA

Oligonucleotides used for off-target effects from the livers of three mutant mice generated by microinjection into one blastomere of two-cell embryos.

Template	Direction	Sequence (5' to 3')
off1	F	TCCTCCATTCAGCCCCTGTA
	R	GTGTTTTTGGAGCAGGCAGG
off2	F	GACAGGACGACGGTGAAGAC
	R	CACTGCCGGTGCTGACTG
off3	F	TAAACCAAGCCCCTTCTGTCAC

	R	CCACACCCAGGGAGAATATGAC
off4	F	TAGGAACATCATCCAGCTAACCAG
	R	TTTCCTTCTAGTCCCAGGACCTCT
off5	F	TGGTTAAATTGTTGTGCTGACCG
	R	AGCATCCGCTCACACTAAGG
off6	F	ACCTCCACTCCCACCTTTCT
	R	TCGTGGGTGTAGCTACCAGT
off7	F	GCTTGAGATATACACGGTCCCA
	R	GAGGTCTGAATCTCACCAGTCA
off8	F	GTCTGGCACACATTGGACCC
	R	TTCAGTTCATCACTCATGTGTCTCA
off9	F	ACACTACCCAGGCTGTAGAC
	R	AGATTTATTTGGCATCTGGCTTCTG
off10	F	ACCTTGTTTGGACTGGGGTC
	R	CAGACAGACACATGCACACG

Gene primers used for *in situ* hybridization

Gene name	Accession no.	Sense primer	Antisense primer	Size of PCR product (bp)
Slc17a5	NM_001276452.1	aaacgacgatgaggagagct	aacgcaacactcaggttcac	162
Virma	NM_001081183.1	tgacaacagagcatatgggga	ttctccctcttcttgcggtt	586

Oligonucleotides used for sequencing of individual clones from RT-PCR product of *Virma* mRNA.

Template	Direction	Sequence (5’ to 3’)
Virma	F	ATCCGAGCGCTGAGCAAAGT
	R	AAACTGGAGCACTTGGTTTGC

Supplementary table 7. Reagents used in this study, related to experimental procedures.

REAGENT	SOURCE	IDENTIFIER
Antibodies		
Rabbit polyclonal anti-SLC17A5 antibody	Thermofisher	Cat# PA5-42456
Rabbit monoclonal anti-Aquaporin 5 antibody	Abcam	Cat# ab92320
Mouse monoclonal anti-TMEM16A	MXB Biotchnologies	Cat# MAB-0335
IgM Antibody (A-7)	Santa Cruz	Cat# sc-373781
C3 Antibody (B-9)	Santa Cruz	Cat# sc-28294
Pacific Blue™ anti-mouse CD4	Biolegend	Cat# 100428
Brilliant Violet 605™ anti-mouse CD3ε	Biolegend	Cat# 100351
PE anti-mouse CD25	Biolegend	Cat# 102008
FITC anti-mouse CD25	Biolegend	Cat# 101907
APC anti-mouse CD127 (IL-7Rα)	Biolegend	Cat# 135012
PE anti-mouse CD152 (CTLA-4) Antibody	eBioscience	Cat# 12-1529-42
PerCP-Cyanine5.5 anti-mouse FOXP3 Antibody	eBioscience	Cat# 45-5773-82
Donkey anti-Mouse IgG (H+L) Highly Cross-Adsorbed Secondary Antibody, Alexa Fluor 488	Invitrogen	Cat# A-21202
Alkaline phosphatase conjugated anti-digoxigenin Fab	Roche	Cat# 14608124
Chemicals, Peptides, and Recombinant Proteins		
Cas9 protein (S. pyogenes)	NEB	Cat# M0386
Q5® Hot Start High-fidelity DNA Ploymerase	NEB	Cat# M0493
mMESSAGE mMACHINE™ T7 ULTRA Transcription kit	Invitrogen	Cat# AM1345
MEGAshortscript™ T7 Transcription kit	Invitrogen	Cat# AM1354
MEGAclear™ Transcription Clean-Up Kit	Invitrogen	Cat# AM1908
RevertAid First Strand cDNA Synthesis Kit	Thermofisher	Cat# K1621
Zymoclean™ Gel DNA Recovery	Zymo Research	Cat# D4007
DNA Clean & Concentrator™-5	Zymo Research	Cat# D4014
Hieff™ PCR Master Mix	Yeasen	Cat# 10102
MALBAC® Single Cell WGA Kit	Yikon	Cat# KT110700110/ YK001A

Ex Taq	TaKaRa	Cat# RR001
Pregnant Mare Serum Gonadotrophin (PMSG)	Prospec	Cat# HOR-272
Human Chorionic Gonadotrophin (hCG)	Prospec	Cat# HOR-250
M2 media	Sigma-Aldrich	Cat# M7167
Mineral oil	Sigma-Aldrich	Cat# M8410
Hyaluronidase	Sigma-Aldrich	Cat# H3506
EmbryoMax® Modified M16 Medium (1X)	Merck	Cat# MR-016-D
Animal Tissues/Cells Genomic DNA Extraction Kit	Solarbio	Cat# D1700
pEASY-T1 Simple Cloning Kit	Transgen	Cat# CT111
RNAprep pure Tissue Kit	Tiangen	Cat# DP431
GoTaq® qPCR Master Mix	Promega	Cat# A6002
Total Nitric Oxide and Nitrate/Nitrite Assay	R&D Systems	Cat# KGE001
Sialic Acid Assay Kit	Abnova	Cat# KA1655
T7 RNA Polymerase	Roche	Cat# 10881767001
DIG RNA Labeling Mix	Roche	Cat# 11277073910
BCIP substrates	Promega	Cat# 0000094915
NBT substrates	Promega	Cat# 0000109902
