# Supplementary material for: Generating viable mice with heritable embryonically lethal mutations using the CRISPR-Cas9 system in two-cell embryos
Source: Nat Commun. 2019 Jun 28;10:2883. doi: 10.1038/s41467-019-10748-2 (PMC6599060; doi:10.1038/s41467-019-10748-2)
Supplement: Supplementary file 3 — Description of Additional Supplementary Files [file 41467_2019_10748_MOESM3_ESM.doc]

**Title:** Supplementary movie 1
**Description:** Phenotype of founder chimeric *Slc17a5* knockout mice generated by microinjecting Cas9 mRNA and sgRNA into zygotes.
