# Supplementary material for: Generating viable mice with heritable embryonically lethal mutations using the CRISPR-Cas9 system in two-cell embryos
Source: Nat Commun. 2019 Jun 28;10:2883. doi: 10.1038/s41467-019-10748-2 (PMC6599060; doi:10.1038/s41467-019-10748-2)
Supplement: Supplementary file 4 — Reporting Summary [file 41467_2019_10748_MOESM4_ESM.pdf]

nature research

Corresponding author(s): Songlin Wang

Reporting Summary

Nature Research wishes to improve the reproducibility of the work that we publish. This form provides structure for consistency and transparency in reporting. For further information on Nature Research policies, see Authors & Referees and the Editorial Policy Checklist.

Statistical parameters

When statistical analyses are reported, confirm that the following items are present in the relevant location (e.g. figure legend, table legend, main text, or Methods section).

n/a	Confirmed	
☐	☒	The exact sample size (*n*) for each experimental group/condition, given as a discrete number and unit of measurement
☐	☒	An indication of whether measurements were taken from distinct samples or whether the same sample was measured repeatedly
☐	☒	The statistical test(s) used AND whether they are one- or two-sided<br>*Only common tests should be described solely by name; describe more complex techniques in the Methods section.*
☒	☐	A description of all covariates tested
☐	☒	A description of any assumptions or corrections, such as tests of normality and adjustment for multiple comparisons
☐	☒	A full description of the statistics including central tendency (e.g. means) or other basic estimates (e.g. regression coefficient) AND variation (e.g. standard deviation) or associated estimates of uncertainty (e.g. confidence intervals)
☐	☒	For null hypothesis testing, the test statistic (e.g. *F*, *t*, *r*) with confidence intervals, effect sizes, degrees of freedom and *P* value noted<br>*Give P values as exact values whenever suitable.*
☒	☐	For Bayesian analysis, information on the choice of priors and Markov chain Monte Carlo settings
☒	☐	For hierarchical and complex designs, identification of the appropriate level for tests and full reporting of outcomes
☒	☐	Estimates of effect sizes (e.g. Cohen's *d*, Pearson's *r*), indicating how they were calculated
☐	☒	Clearly defined error bars<br>*State explicitly what error bars represent (e.g. SD, SE, CI)*

Our web collection on statistics for biologists may be useful.

Software and code

Policy information about availability of computer code

Data collection	No software was used.
Data analysis	CRISPOR software for potential off-target sites for sgRNA. Fiji and Image J softwares for image analysis. CRISPRko software for sgRNA selection. Graph Prism (v.7) software for statistical analysis. Flowjo V10 for flow cytometry analysis.

For manuscripts utilizing custom algorithms or software that are central to the research but not yet described in published literature, software must be made available to editors/reviewers upon request. We strongly encourage code deposition in a community repository (e.g. GitHub). See the Nature Research guidelines for submitting code & software for further information.

Data

Policy information about availability of data

All manuscripts must include a data availability statement. This statement should provide the following information, where applicable:

- Accession codes, unique identifiers, or web links for publicly available datasets
- A list of figures that have associated raw data
- A description of any restrictions on data availability

Whole-genome sequencing data from this study are available through the NCBI Sequence Read Archive under accession number PRJNA543729. The RNA high-throughput sequencing data reported in this paper has been deposited in the NCBI Sequence Read Archive (accession no. PRJNA543953).

Field-specific reporting

Please select the best fit for your research. If you are not sure, read the appropriate sections before making your selection.

☒ Life sciences ☐ Behavioural & social sciences ☐ Ecological, evolutionary & environmental sciences

For a reference copy of the document with all sections, see nature.com/authors/policies/ReportingSummary-flat.pdf

Life sciences study design

All studies must disclose on these points even when the disclosure is negative.

Sample size	The sample size used in this study is determined based on experience according to similar literatures. This sample size is determined with the expense of data collection, and the need to have sufficient statistical power.
Data exclusions	No data were excluded from the analyses.
Replication	All measurements were repeated for at least three times. The reproducibility of the experimental findings can be successfully replicated according to the Methods and Supplementary Methods.
Randomization	Animals were randomly allocated into experimental groups.
Blinding	We were blinded to group allocation during data collection and/or analysis

Reporting for specific materials, systems and methods

Materials & experimental systems

n/a	Involved in the study	
☒	☐	Unique biological materials
☐	☒	Antibodies
☒	☐	Eukaryotic cell lines
☒	☐	Palaeontology
☐	☒	Animals and other organisms
☒	☐	Human research participants

Methods

n/a	Involved in the study	
☒	☐	ChIP-seq
☐	☒	Flow cytometry
☒	☐	MRI-based neuroimaging

Antibodies

Antibodies used	IHC:Rabbit polyclonal anti-SLC17A5 antibody (Thermofisher, PA5-42456), Rabbit monoclonal anti-Aquaporin 5 antibody (Abcam,ab92320), Mouse monoclonal anti-TMEM16A (MXB Biotchnologies,MAB-0335), Alkaline phosphatase conjugated anti-digoxigenin Fab (Roche,14608124).IHF: IgM Antibody (A-7) (Santa Cruz, sc-373781), C3 Antibody (B-9) (Santa Cruz, sc-28294), Donkey anti-Mouse IgG (H+L) Highly Cross-Adsorbed Secondary Antibody, Alexa Fluor 488 (Invitrogen, A-21202). FACS: Pacific Blue™ anti-mouse CD4 (Biolegend, 100428), Brilliant Violet 605™ anti-mouse CD3ε (Biolegend, 100351), PE anti-mouse CD25 (Biolegend,102008), FITC anti-mouse CD25 (Biolegend, 101907), APC anti-mouse CD127 (IL-7Rα) (Biolegend, 135012), PE anti-mouse CD152 (CTLA-4) Antibody (eBioscience, 12-1529-4), PerCP-Cyanine5.5 anti-mouse FOXP3 Antibody (eBioscience, 45-5773-82).
Validation	all antibodies were used for IHC, IHF and FACS.

Animals and other organisms

Policy information about studies involving animals; ARRIVE guidelines recommended for reporting animal research

Laboratory animals	C57BL/6J,Cat#: 219, Beijing Vital River Laboratory Animal Technology Co., Ltd.
Wild animals	The study did not involve wild animals.
Field-collected samples	The study did not involve samples collected from the field.

Flow Cytometry

Plots

Confirm that:

☒ The axis labels state the marker and fluorochrome used (e.g. CD4-FITC).

☒ The axis scales are clearly visible. Include numbers along axes only for bottom left plot of group (a 'group' is an analysis of identical markers).

☒ All plots are contour plots with outliers or pseudocolor plots.

☒ A numerical value for number of cells or percentage (with statistics) is provided.

Methodology

Sample preparation	Mice were sacrificed by cervical dislocation. A single-cell suspension was achieved by passing the spleen through a 70 µm nylon cell strainer (BD Biosciences). The single cell suspension was washed in PBS and resuspended in erythrocyte lysis buffer (Qiagen) to remove the red blood cells.
Instrument	FACSAriaII (BD Biosciences)
Software	FlowJo software (Tree Star)
Cell population abundance	The proportion of post-sort CD4+ CD25+CD127- Treg cells is more than 95% in lymphocytes.
Gating strategy	Lymphocytes were analysed by flow cytometry, gating strategy on lymphocytes, single, CD4+ cells. Treg cells were then identified as CD25+CD127- or Foxp3+, while CTLA4+ Tregs were further gated from sorted CD4+CD25+CD127- population.

☒ Tick this box to confirm that a figure exemplifying the gating strategy is provided in the Supplementary Information.
